# Duration Matters: Tailoring Antibiotic Therapy for Ventilator-Associated Pneumonia

**DOI:** 10.3390/antibiotics15010034

**Published:** 2026-01-01

**Authors:** Tim Rahmel, Isabella Traut, Lars Bergmann, Maria Panagiota Almyroudi, Barbara Tamowicz, Priyam Varma, Despoina Koulenti, Antonios Katsounas

**Affiliations:** 1 Department of Anesthesiology, Intensive Care Medicine and Pain Therapy, Knappschaft Kliniken University Hospital Bochum, Ruhr-University Bochum, 44892 Bochum, Germany; 2 Division of Clinical Infectious Diseases and Internal Critical Care, Department of Internal Medicine, Knappschaft Kliniken University Hospital Bochum, Ruhr-University Bochum, 44892 Bochum, Germany; 3 Department of Emergency Medicine, Attikon University Hospital, National and Kapodistrian University of Athens, 12462 Athens, Greece; mariotaalm@yahoo.gr; 4 Faculty of Health Sciences, Poznań University of Medical Sciences, 60-806 Poznań, Poland; tamowicz@esculap.pl; 5 Critical Care Department, King’s College Hospital NHS Foundation Trust, London SE5 9RS, UK; priyam.varma1@nhs.net; 6 UQ Centre for Clinical Research (UQCCR), The University of Queensland, Brisbane 4072, Australia; 7 School of Medicine, University of Thessaly, 41500 Larissa, Greece; 8 School of Health Sciences, University of Ioannina, 45110 Ioannina, Greece

**Keywords:** ventilator-associated pneumonia, antibiotic duration, antimicrobial stewardship, procalcitonin, *Pseudomonas* spp., non-fermenting Gram-negative bacilli, intensive care unit

## Abstract

Ventilator-associated pneumonia (VAP) remains the most frequent ICU-acquired infection and a major driver of antimicrobial exposure. Historically, clinicians treated patients for 10–14 days or longer, particularly when multidrug-resistant organisms were suspected. Current evidence from randomized trials and meta-analyses now supports shorter-course therapy (~7 days) for most immunocompetent patients with VAP who demonstrate clinical improvement. Mortality and treatment failure are not increased when compared with longer regimes. The REGARD-VAP trial demonstrated the non-inferiority of individualized ≤7-day therapy compared with conventional longer courses. This remained true even in cohorts rich in non-fermenting Gram-negative bacilli (NF-GNB) and carbapenem-resistant organisms while markedly reducing antibiotic-related toxicity. North American and European guidelines recommend 7–8 days as the default duration, with individualized extension for slow clinical response, bacteremia, uncontrolled foci, or profound immunosuppression. Additionally, biomarker-guided discontinuation, particularly serial procalcitonin (PCT), may reduce antibiotic days when used to enrich clinical assessment. This narrative review synthesizes guideline recommendations, trial evidence, biomarker-guided stewardship, and pathogen- and patient-specific scenarios to provide a practical framework for intensivists: treat until infection is controlled and the patient is improving, usually about 1 week, and extend therapy only with clear justification.

## 1. Introduction

Ventilator-associated pneumonia (VAP) is the most common intensive care unit (ICU)-acquired infection, occurring in an estimated 10–20% of patients on mechanical ventilation [1]. VAP is associated with significant morbidity and mortality, including prolonged duration of mechanical ventilation, increased ICU and hospital length of stay, all-cause mortality rates of 20–50%, and an attributable mortality of 13%. Even higher attributable mortality rates have been reported for *Pseudomonas aeruginosa* VAP [1,2,3,4,5,6]. Beyond its direct impact on patients, VAP contributes substantially to ICU antibiotic consumption and antimicrobial resistance pressure [7]. Historically, treatment durations for VAP were prolonged (typically 10–14 days or longer), driven by concerns about undertreatment and relapse, particularly in infections caused by multidrug-resistant (MDR) pathogens [8]. This conservative approach persisted for decades, reflecting limited evidence and considerable uncertainty regarding optimal treatment duration. Longer courses were often selected for challenging pathogens such as *P. aeruginosa*—where recurrence risk is a concern—and for MDR organisms, for which guidelines recommend individualized duration guided by clinical, biomarker, and imaging findings [1,2,8,9].

In recent years, however, a paradigm shift has emerged. Growing evidence from randomized controlled trials (RCTs) and meta-analyses, including the pivotal REGARD-VAP trial [10] published in 2024, suggests that shorter antibiotic courses of approximately 7 days are sufficient for most immunocompetent patients with VAP, without compromising clinical outcomes. Shorter treatment durations of 7–8 days potentially yield substantial benefits, including reduced antibiotic exposure, fewer drug-related adverse events, lower risk of *Clostridioides difficile* infection, and less selective pressure for resistance [11]. Reflecting this evidence, major international guidelines have shifted to recommend 7–8-day regimens for VAP, whereas earlier guidelines from the 1990s–2000s often endorsed 10–14 days [1,2]. In immunocompetent patients, shorter antibiotic courses achieve clinical outcomes comparable to longer regimens while reducing antibiotic exposure and treatment-related toxicity. Importantly, patients with significant immune dysfunction were largely excluded from the randomized trials underpinning these conclusions or represent only a small minority within these studies. As a result, the applicability of short-course therapy to immunocompromised or immunoparalytic patients remains uncertain and requires individualized clinical decision-making beyond the scope of this review. Nevertheless, questions remain in immunocompetent patients regarding specific VAP scenarios in which longer therapy may still be justified—for example, certain high-risk pathogens, such as non-fermenting Gram-negative bacilli (NF-GNB), or cases with slow clinical resolution. In these situations, clinicians must carefully balance the potential risk of relapse against the well-established harms of unnecessary prolonged antibiotic exposure.

This narrative review examines the current evidence and recommendations on antibiotic duration in VAP. We first summarize key guideline recommendations and then review the clinical trial data that underpins these recommendations and may modify future expert consensus. We discuss the integration of biomarkers (especially procalcitonin) and other stewardship tools to individualize therapy duration. Additionally, we review special circumstances, such as infections caused by MDR pathogens or complicated VAP (e.g., cases accompanied by bacteremia), which may warrant an extended antibiotic duration. An evolving theme is that treatment duration should be guided more by patient-specific factors, including clinical response and source control where appropriate, rather than by a fixed notion that certain organisms always require longer therapy. The overarching goal is to treat until the infection is controlled and the patient is demonstrably improving, while avoiding unnecessary prolongation of therapy.

## 2. Guideline Recommendations on Antibiotic Duration

Over the past decade, VAP management guidelines have increasingly endorsed shorter antibiotic courses to optimize efficacy while minimizing toxicity and development of resistance [1,11,12]. Table 1 highlights major guideline recommendations on VAP treatment duration. In general, 7 days of treatment is now recommended as adequate for most patients who demonstrate clinical response to treatment and is referred to as “short” in this review, although it now represents the current “standard” compared with the “long” courses of 10–14 days, with extension reserved for selected cases. Despite minor variations, these guidelines uniformly pivot away from the old 14-day paradigm. They stress that routine prolonged therapy provides no advantage in VAP and may increase adverse effects. Longer durations are not justified solely by the organism. For example, infection with *Pseudomonas* or methicillin-resistant *Staphylococcus aureus* (MRSA) does not, by itself, mandate treatment beyond 7 days if the patient is clinically improving. Instead, any extension beyond approximately 1 week should be guided by specific clinical circumstances, as discussed later. Table 1 summarizes the duration recommendations from major guidelines.

Current guidelines emphasize that close patient monitoring and proper clinical judgment are essential. If a patient with VAP shows clear clinical improvement by days 5–7, a total antibiotic course of 7 days is generally sufficient in most cases. Conversely, if the patient is not improving, clinicians should not continue antibiotics indefinitely but instead re-evaluate the situation, as discussed in a later section, on when to extend therapy. This shift in guidelines is clear, yet clinician adoption has been gradual. Recent studies found nearly half of ICU physicians still favor >7-day courses for antibiotic treatment of VAP, indicating ongoing gaps between evidence and clinical practice [16]. The following sections review the key evidence underpinning the rationale for shorter durations.

## 3. Clinical Evidence from Trials and Meta-Analyses

Multiple clinical trials over the past two decades have directly examined antibiotic duration in VAP. These studies, along with the meta-analyses summarizing their results, strengthen the evidence that shorter courses are generally safe and effective. The following section outlines the major trials, meta-analyses, and other recent landmark studies on VAP treatment duration.

### 3.1. Randomized Controlled Trials

The PneumA Trial [9]: This French multicenter RCT was the first pivotal study to compare short- and long-course therapy in VAP. In total, 401 patients with microbiologically confirmed VAP were randomized to receive 8 days versus 15 days of antibiotics. The results showed no significant differences in key outcomes between the short and long courses: 28-day mortality was approximately 18%, and clinical recurrence of pneumonia was 27–29% in both groups. Patients in the 8-day arm had 5 antibiotic-free days on average, more than those in the 15-day arm (13.1 vs. 8.7 days). Importantly, short-course therapy was not associated with increased mortality or ICU length of stay. The only notable difference was observed in a subset analysis of *P. aeruginosa* VAP. Here, recurrence of infection with *Pseudomonas* was higher in the 8-day group compared to the 15-day arm (40.6% vs. 25.4%). However, this did not translate into higher mortality among patients with *Pseudomonas* VAP, as survival was similar in both arms. Furthermore, among those who relapsed, the short-course patients had fewer MDR organisms emerge (42% vs. 62%). In conclusion, an 8-day antibiotic course was as effective as 15 days on VAP overall, with a higher relapse risk in *Pseudomonas* but no impact on patient mortality. This trial established 7–8 days as a viable strategy and helped to shape subsequent guideline changes.

iDIAPASON Trial [17]: Given concerns about *Pseudomonas* from the PneumA Trial [9], Bouglé and colleagues conducted a targeted RCT of VAP due to *P. aeruginosa*. Published in 2022, this French study randomized 186 patients with *Pseudomonas* VAP to 8 days versus 15 days of therapy. The trial was discontinued early due to slow enrollment, and it did not meet non-inferiority criteria. In the 8-day group, the composite outcome of mortality or recurrence was 35.2% vs. 25.5% in the 15-day group (difference, ~9.7%; 90% CI −1.9% to 21%). Recurrence of *Pseudomonas* VAP nearly doubled with short therapy (17% vs. 9.2%). However, no significant difference in mortality or ICU length of stay was found between the groups. This suggests that while there were more relapses with 8-day therapy, these recurrences were managed without affecting the overall clinical outcome. The authors concluded that while 8 days may carry a higher relapse risk in *Pseudomonas* VAP, the study was underpowered, and no survival benefit of 15 days was demonstrated. This aligns with Chastre’s finding that extended courses reduce microbiologic relapse but not overall mortality [9]. Bouglé et al. recommend caution and possibly longer therapy in certain cases of *Pseudomonas* but emphasize that many *Pseudomonas* VAP cases can be successfully treated in 7–8 days if patients display sustained clinical improvement.

REGARD-VAP Trial [10]: The REGARD-VAP trial is a recent randomized controlled study that provides the strongest evidence to date supporting shorter antibiotic courses in VAP. This multicenter trial (39 ICUs in Thailand, Singapore, and Nepal) randomized 461 patients with VAP to an individualized short-course strategy (≤7 days) versus usual care (≥8 days, often 10–14 days). In the short-course arm, antibiotics were discontinued once patients were clinically stable (≥48 h afebrile and hemodynamically stable), with a minimum of 3 days for culture-negative VAP and 5 days for microbiologically confirmed cases. More than half of the enrolled patients had infections caused by NF-GNB, primarily *Pseudomonas* or *Acinetobacter*, and one-third had carbapenem-resistant pathogens. Thus, the study addressed prior criticisms that short-course trials underrepresented difficult-to-treat organisms. The primary composite outcome (60-day mortality or VAP recurrence) was nearly identical between groups (41% in the ≤7-day arm vs. 44% in the ≥8-day arm), meeting the predefined 12% non-inferiority margin. Outcomes were consistent across subgroups, including those with NF-GNB and carbapenem-resistant isolates, and even among culture-negative pneumonias. Shorter therapy markedly reduced antibiotic exposure (median 6 vs. 14 days for the initial VAP episode) and antibiotic-related toxicity, with nephrotoxicity decreasing from 35% to 5%—an absolute reduction of ~30%. Other adverse events (e.g., hepatotoxicity, *C. difficile*) were also less frequent, without prolongation of ICU stay, ventilation duration, or increased complications. Although antibiotic therapy was re-initiated in approximately one-third of patients in the short-course arm, documented recurrence of VAP accounted for only around half of these cases. This suggests that many cases of re-initiation of antibiotics reflected precautionary practice rather than true relapse. Mortality remained unaffected, reinforcing that early discontinuation appeared safe in this context.

Nevertheless, the REGARD-VAP trial has important limitations. It should also be noted that the definitions of “short” and “long” antibiotic therapy differ substantially across the key trials informing current practice. Most randomized trials and meta-analyses define short-course therapy as 7–8 days and long-course therapy as 10–15 days. In contrast, the REGARD-VAP trial applied a pragmatic, response-adapted strategy, allowing antibiotic discontinuation after a minimum of 3 days for culture-negative cases and 5 days for microbiologically confirmed VAP once predefined clinical stability criteria were met. However, concerns arise regarding patients treated for only 3 days, as they may not have had true VAP, which could artificially inflate the apparent success rate of short therapy. The trial’s inclusion criteria relied on clinical definitions, which lack specificity for bacterial pneumonia and may overestimate infection in patients with non-bacterial conditions. Additionally, the open-label design may have led to selection bias, as clinicians may have favored patients already perceived to be improving, thus skewing results toward non-inferiority.

Although conducted across three countries, most patients were recruited in Thailand, which may limit generalizability to high-resource ICUs with different staffing models, diagnostic tools (e.g., routine bronchoalveolar lavage and biomarker-guided decisions), and patient populations. The pragmatic, open-label design introduces potential for performance and detection bias, including differential ancillary care or subjective outcome assessment. Furthermore, some patients treated for only 3 days may not have had true VAP but rather colonization or ventilator-associated tracheobronchitis, potentially inflating apparent success rates. Selection bias is also possible if enrollment favored cases where clinicians were already comfortable with shorter therapy, thereby biasing results toward non-inferiority.

Taken together, REGARD-VAP supports the safety and efficacy of a shorter, individualized antibiotic duration strategy, even in cohorts with a high prevalence of MDR pathogens, but its findings should be interpreted with appropriate caution. Broader confirmation in diverse, high-income settings and with more rigorous diagnostic and biomarker integration will be essential before universal adoption.

### 3.2. Meta-Analysis

Two notable meta-analyses published in 2023 aggregated existing trials to compare short versus long antibiotic courses in VAP.

Daghmouri et al. [18]: This systematic review/meta-analysis combined data from five RCTs (total *n* = 1069 VAP patients) comparing ≤8 days vs. ≥10–15 days. The results showed no significant difference in VAP recurrence or relapse rates between short and long therapies. The odds ratio for recurrence showed a non-significant trend toward more recurrences with short therapy (OR: 1.48, 95% CI ~0.96–2.28, *p* = 0.08). Similarly, risk of relapse was not significantly increased (*p* = 0.09). In the NF-GNB subgroup (3 trials, ~330 patients), short courses had higher recurrence odds (OR~1.9) that just reached the threshold of statistical significance (*p* = 0.05). This suggests an approximately 2-fold higher recurrence risk in *Pseudomonas* and *Acinetobacter* VAP when treated with a short course, consistent with the findings from Chastre [9] and Bouglé [17]. However, 28-day mortality did not differ between groups, and neither did other outcomes such as ventilator days, ICU length of stay, or extra-pulmonary infections. Short-course patients unsurprisingly had more antibiotic-free days, and the incidence of emerging MDR infections during ICU stay was not affected. If anything, prolonged antibiotics would be expected to foster more MDR infections, a risk that short courses mitigate. The authors cautioned that outcome definitions varied across studies and that confidence intervals were somewhat wide, but they overall concluded that 7–8 days is generally as effective as 10–15 days for VAP. They recommended focusing on careful monitoring to detect any recurrences early, rather than reflexively treating everyone for 2 weeks.

Valladares et al. (2025) [19]: This meta-analysis on the use of inhaled antibiotics for VAP prevention demonstrated that inhaled antibiotics significantly reduced the incidence of VAP (RR = 0.67; 95% CI: 0.58–0.77). This outcome is consistent with findings supporting the role of inhaled antibiotics in reducing infection rates but emphasizes that no significant reduction in ICU mortality was observed (RR = 0.92; 95% CI: 0.79–1.06). The findings reinforce the utility of inhaled antibiotics for VAP prevention but caution against assuming survival benefit from this intervention.

Cheema et al. [11]: This meta-analysis included six RCTs (*n* = 1274) comparing short (~7–8 days) versus long (~10–15 days) regimens. Overall, the findings were consistent with the meta-analysis of Daghmouri et al. [18], with no significant differences in mortality, clinical cure, or length of stay between short and long courses. However, Cheema et al. did report a statistically significant increase in relapse rates for NF-GNB VAP with short courses. This suggests that in pooled data, short therapy (7–8 days) may lead to more pseudomonal relapses, which is consistent with previous concerns. Despite this, there was no difference in clinical outcomes or mortality, implying that relapses could be successfully re-treated without an impact on prognosis. The authors rated the evidence quality as low (due to trial biases and heterogeneity) but concluded that routine prolonged therapy is not warranted, and short courses should be used with the understanding that a minority of patients, specifically with certain pathogens, may need retreatment. The slight uptick in recurrence must be weighed against the benefits of minimizing unnecessary antibiotic exposure in the majority of patients.

In essence, these meta-analyses reinforce the message: a 7- to 8-day course is appropriate for most VAP cases, with outcomes equivalent to longer regimens that were traditionally used in the past. Although a small increase in recurrence of NF-GNB infections is acknowledged, this does not translate into higher mortality and can be managed with close clinical follow-up. These findings echo the RCT of Chastre and colleagues [9] and provide further support for short-course therapy strategies. An earlier Cochrane Review in 2015 similarly found no significant difference in outcome between short versus long therapy aside from *Pseudomonas* relapse [20].

### 3.3. Further Studies

Beyond RCTs, real-world data has also emerged supporting shorter therapy even in challenging scenarios. For example, Truong et al. examined outcomes in ICU patients with MDR *P. aeruginosa* pneumonia (including VAP) treated for ≤8 days vs. >8 days [21]. They found no difference in clinical success or mortality between short and long treatment courses, though recurrence was slightly higher with the short-course group. However, these recurrences were manageable with retreatment. Another analysis by Al-Musawa et al. [22] focusing on MDR *P. aeruginosa* HAP/VAP likewise reported no mortality benefit for extended therapy beyond 7 days. These findings underpin that even for notoriously difficult pathogens, longer treatment durations do not improve survival. In contrast, once the patient is improving, continuing antibiotics generally increases avoidable risks without providing additional benefits.

Nemet et al. (2025) [23] further contribute by emphasizing the impact of structural and system factors in the risk of VAP, particularly in low- and middle-income countries. Their study shows a 2-fold increase in VAP risk in low- and middle-income-country ICUs, with key associations including prolonged mechanical ventilation and staffing ratios. This research underscores the need for addressing healthcare infrastructure disparities in order to reduce VAP risks, highlighting that systems-level interventions are as crucial as clinical decisions in managing VAP.

An important limitation when interpreting recurrence, relapse, or treatment failure across VAP trials is the substantial heterogeneity in outcome definitions. Terms such as recurrence, relapse, reinfection, and treatment failure are often used inconsistently and are operationalized differently across studies, ranging from microbiological re-isolation to clinically driven antibiotic re-initiation. Distinguishing true relapse of the index infection from new infection, recolonization, or non-infectious clinical deterioration is particularly challenging in mechanically ventilated patients. This definition variability complicates comparison across trials and may inflate pooled recurrence estimates in meta-analyses. The recently proposed RECUVAP consensus represents an important step toward harmonizing outcome definitions in VAP research, aiming to improve the comparability and interpretability of future trials [24]. Until such standardized definitions are consistently applied, recurrence-related outcomes should be interpreted with caution, particularly when used to justify prolonged antibiotic durations.

In summary, the weight of clinical evidence supports short-course therapy (~7 days) for VAP in most cases. Longer treatment does not improve survival or overall cure rates, although it may reduce the likelihood of microbiologic recurrence in some *Pseudomonas* or *Acinetobacter* cases. However, those recurrences are usually treatable and not associated with worse outcomes [9,17,20]. In contrast, prolonged therapy universally increases antibiotic exposure and the associated risks [11,18]. The key stewardship message is clear: for most VAP patients who are responding to appropriate therapy, antibiotics can be safely discontinued after approximately 1 week. The following sections will discuss how clinicians can implement this approach effectively, using biomarkers, monitoring, and individualized considerations, as well as which scenarios might warrant deviations from the 7-day standard.

## 4. Biomarkers and Diagnostic Stewardship for Guiding Duration

One strategy to individualize and potentially shorten antibiotic duration in VAP is the use of biomarkers to guide cessation versus continuation decisions [25]. Procalcitonin (PCT), in particular, has been studied extensively as a tool to gauge infection resolution and support early discontinuation of antibiotics [26,27,28]. The rationale is that biomarkers provide objective data on the host’s inflammatory response, supplementing clinical judgment when deciding if it is safe to stop therapy. This section reviews the role of PCT and other biomarkers and how they fit into an antibiotic stewardship approach.

Major guidelines have taken different stances on incorporating PCT into VAP management. The IDSA/ATS 2016 guideline suggests that serial PCT measurements, alongside clinical criteria, can be used to support discontinuation of antibiotics, especially if the planned course would otherwise exceed 7 days [1]. In practice, this means if a patient has received approximately 1 week of appropriate therapy and is improving, a low or rapidly declining PCT can further strengthen the decision to stop antibiotics earlier rather than arbitrarily continuing to 10–14 days. In contrast, the ERS/ESICM 2017 European guidelines advise against routine PCT to guide therapy duration if a 7- to 8-day course is already planned [2]. Their rationale is that when a short course is defined a priori, the addition of PCT provides limited incremental benefit, as clinicians should primarily focus on clinical improvement. However, in scenarios where one is unsure about stopping at day 7, PCT may be helpful. The new German 2024 guidance takes a middle ground: it acknowledges that a PCT-based algorithm may be helpful in ambiguous cases, for instance, if a patient’s clinical response is equivocal around day 7 [15]; trending PCT might inform whether to stop or continue treatment. Similarly, the Chinese 2018 guidelines list dynamic PCT monitoring as an adjunct (Level II evidence) for deciding duration [14]. All guidelines agree on one point: clinical improvement is the primary determinant of duration, and biomarkers should complement, but not replace, bedside assessment. In summary, PCT is recognized as a potentially useful tool; however, recommendations regarding its routine implementation vary, as it fundamentally requires a seamless integration into a clinically based decision-making algorithm. Institutions with strong stewardship programs often integrate PCT into an “antibiotic stop” protocol around day 5–7 of treatment [29].

In this context, various PCT-guided algorithms have been evaluated, primarily in non-VAP infections such as community-acquired or ventilator-associated lower respiratory tract infections [26,28,29]. However, evidence specifically supporting PCT-guided duration adjustment in VAP remains limited, and further data is needed. Commonly, thresholds used to stop antibiotic treatment in severe infections (including VAP) are an absolute PCT <0.5 ng/mL or a ≥80% decline from the peak PCT value. Achieving either of those targets suggests that the bacterial infection is substantially controlled, and antibiotics can be stopped even if the originally intended course is not completed. For example, a patient who had a peak PCT of 8 ng/mL that drops to 0.8 ng/mL (90% reduction) after 5–6 days of treatment likely has minimal ongoing infection. These cutoff criteria were used in several landmark trials:

- The PRORATA trial [26], conducted on ICU patients with various types of infection, demonstrated that using a PCT-guided algorithm (with thresholds of an absolute PCT value < 0.5 ng/mL or a ≥80% decline from peak levels to discontinue antibiotics) reduced antibiotic exposure by approximately 23% without increasing mortality or treatment failure.
- A large Dutch ICU study by de Jong et al. [30], focusing on patients with sepsis (including VAP), found that the PCT-guided group (using the same discontinuation thresholds of an absolute PCT value < 0.5 ng/mL or a ≥80% decline from peak levels) had shorter antibiotic courses and slightly lower mortality compared with standard care.
- A 2017 meta-analysis by Schuetz et al. [31] confirmed that PCT-guided antibiotic stewardship in respiratory infections leads to reduced antibiotic exposure and lower risk of antibiotic-related side effects, with no increase in morbidity or mortality. However, VAP accounted for only 6% of the cases in the included studies.
- Importantly for VAP, Stolz and colleagues conducted an RCT [32] on 101 VAP patients. This RCT specifically demonstrated that a PCT-guided strategy (absolute PCT value < 0.5 ng/mL or a ≥80% decline from peak levels) can safely shorten therapy duration in VAP. In this study, the PCT group had more antibiotic-free days alive by day 28 (median 13 days vs. 9.5 days in controls). This represented a 27% reduction in total antibiotic duration in the PCT group. Remarkably, there were no differences in clinical outcomes: mechanical ventilation days, ICU length of stay, and 28-day mortality were similar between PCT-guided and standard groups. Thus, PCT guidance achieved a reduction in treatment length without harming patients.
- The REGARD-VAP trial [10] discussed earlier, incorporated PCT use into its protocol—in the short-course arm, one of the criteria for antibiotic cessation was an 80% decline in PCT (alongside clinical criteria). Importantly, the REGARD-VAP trial did not incorporate rapid molecular diagnostic platforms nor perform analyses correlating specific resistance genes with clinical trajectories, relapse timing, or the need for antibiotic retreatment. Subgroup analyses were based on conventional culture-derived phenotypic resistance categories (e.g., NF-GNB and carbapenem-resistant organisms), and outcomes were assessed at the clinical rather than molecular level. Therefore, conclusions from REGARD-VAP should not be extrapolated to genotype-driven treatment duration strategies. Therefore, REGARD’s success in the short arm might have been partly enabled by PCT guidance.

**Figure 1:**
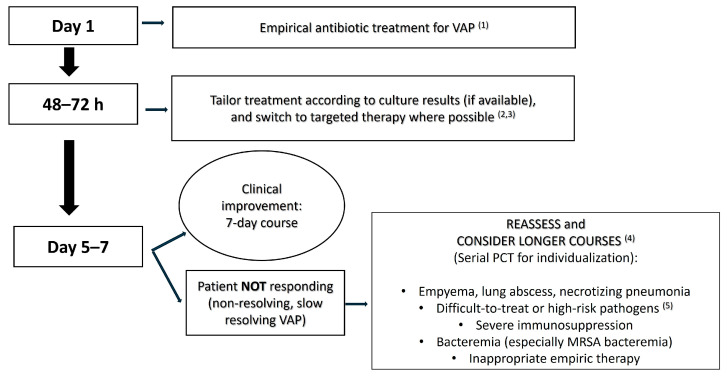
Clinical algorithm for antibiotic discontinuation in VAP. h: hours; MRSA: methicillin-resistant *Staphylococcus aureus*; NF-GNB: non-fermenting Gram-negative bacilli; PCT: procalcitonin. ^(1)^ Rapid molecular diagnostics may facilitate early targeted therapy, leading to optimal antibiotic coverage and early discontinuation. ^(2)^ If patient stable, cultures negative, and VAP diagnosis improbable, consider stopping antibiotics. ^(3)^ If the antibiotic spectrum is appropriate, but the patient is not improving, assess antibiotic dosing, and consider imaging and/or bronchoscopy and may repeat cultures. ^(4)^ Re-assess for appropriate antibiotic choice and dosing, complication, source control if needed, other coexisting infection focus, alternate diagnosis—repeat cultures, imaging, and/or bronchoscopy. ^(5)^ For example, *Pseudomonas aeruginosa*, *Acinetobacter baumannii*, MRSA, NF-GNB.

It is important to recognize that relying on PCT alone, without careful clinical evaluation, is not advisable. PCT levels can be influenced by factors like renal impairment (impaired creatinine clearance can cause PCT to remain elevated) or by non-infectious inflammation [33]. For instance, trauma or surgery can cause moderate PCT elevation [34,35]. A PCT algorithm performs best when embedded within a robust clinical decision-making framework that integrates the patient’s symptoms, physical findings, and overall clinical trajectory. In this integrated context, PCT can provide substantial added value, particularly in situations where the clinical decision to continue or discontinue antibiotics around day 5–7 remains uncertain. Therefore, PCT should augment but not override clinical judgment (Figure 1).

Besides procalcitonin, researchers have explored other biomarkers and tools:

- C-reactive protein (CRP): CRP is a widely available acute-phase reactant [36]. The UK ADAPT-Sepsis trial tested CRP-guided antibiotic duration versus PCT-guided and standard care in sepsis [37]. It found that PCT guidance reduced antibiotic days (by ~1 day) compared to the standard, whereas CRP guidance did not significantly reduce duration. Thus, CRP appears less useful than PCT for this purpose, possibly reflecting its lower specificity and slower decline.
- Clinical Pulmonary Infection Score (CPIS): This is a clinical composite score (fever, leukocytes, oxygenation, secretions, and culture results) sometimes used to assess pneumonia probability. Singh et al. [38] effectively used CPIS as a decision tool to shorten therapy in low-probability cases—if CPIS remained low after 3 days, antibiotics were stopped. While CPIS is not a biomarker per se, it is a clinical algorithm that can aid diagnostic stewardship. Current guidelines recommend against the use of CPIS for VAP diagnosis [1]. However, the German guidelines underscore the principle of re-evaluating at 48–72 h and discontinuing antibiotics if clinical evidence of pneumonia is lacking [15].
- Several other markers have been studied to improve antibiotic decision-making, but none have been validated as a tool to help tailor antibiotic duration in VAP [39,40]. One ICU study found that serial IL-6 monitoring might allow for shorter pneumonia treatment, but the results were not statistically significant [41]. Soluble triggering receptor on myeloid cells and pro-adrenomedullin are among experimental biomarkers, but none are in routine use for guiding duration yet [40].
- Rapid diagnostics and scoring tools: While not a serum biomarker, molecular diagnostics such as the FilmArray Pneumonia Panel offer rapid identification of common respiratory pathogens and resistance genes directly from lower respiratory tract samples [42,43,44]. By providing results within hours, these tools can facilitate earlier targeted de-escalation or help confirm pathogen identity, thereby supporting more individualized decisions about treatment duration rather than its initiation. However, important limitations must be acknowledged. A negative FilmArray result does not rule out infection since the panel covers a limited range of organisms and may miss less common or emerging pathogens [43,45]. Moreover, recent data suggests that repeated testing during therapy does not correlate with clinical outcomes or reduced duration, highlighting that interpretation must always occur within the full clinical context [45]. Beyond microbiological tools, imaging modalities such as lung ultrasound (LUS) are being evaluated to monitor pneumonia resolution. The proposed “CPIS-PLUS” concept—combining the Clinical Pulmonary Infection Score with PCT trends and LUS findings—was designed to enhance treatment assessment response and potentially guide safe discontinuation rather than dictate antibiotic initiation [46,47]. Together, these emerging diagnostic-stewardship tools hold promise for refining decisions on how long to continue antibiotics in VAP, ensuring therapy is neither unnecessarily prolonged nor prematurely curtailed.

In practice, many ICUs now perform an “antibiotic time-out” around day 2–3 of treatment, where cultures and patient status are reviewed [48]. If cultures are negative and the patient is at low risk, antibiotics should be considered for discontinuation to avoid treating a non-existent infection [49]. A serum PCT level of less than 0.25 ng/mL can encourage early antibiotic cessation around day 3 [32,50,51]. However, for VAP, efforts to personalize treatment durations are hindered by the lack of VAP-specific biomarkers that reliably guide antibiotic therapy duration. Existing systemic biomarkers, such as PCT and CRP, do not specifically correlate with pneumonia and may not normalize promptly or predict clinical outcomes effectively, even when patients have clinically improved. This limits their utility in guiding antibiotic therapy decisions for VAP. While PCT may be used to guide decisions about antibiotic cessation, its evidence-based application is primarily derived from community-acquired pneumonia (CAP) settings and does not hold the same proven relevance for VAP. Clinical protocols may include thresholds, such as “If by day 7 the patient is clinically improving and PCT has dropped by more than 80%, plan to stop antibiotics by day 7” [1]. However, this approach, while useful in CAP, is not yet supported by robust evidence for VAP, and such biomarkers should always be used within a broader clinical context, rather than as sole determinants for treatment duration. In VAP, biomarkers like PCT, while helpful in some scenarios, should be considered as part of a broader diagnostic and clinical evaluation rather than a standalone evidence-based tool for determining therapy length [52,53].

Albin et al. (2025) [53] highlight that while biomarkers reflect systemic inflammation, they do not necessarily provide insight into the host and pathogen response to pneumonia treatment itself. This limitation restricts their role in guiding VAP-specific antibiotic duration strategies. Their study calls for a more nuanced approach to VAP diagnosis, where biomarkers are integrated into a broader diagnostic stewardship strategy that complements clinical judgment and helps inform more individualized treatment decisions. This shift toward a comprehensive, tailored approach to VAP management underscores the importance of biomarker-guided stewardship, which, when combined with other diagnostic tools and daily evaluations, can help optimize antibiotic use by ensuring treatment is prescribed for the minimum necessary duration.

In summary, biomarker-guided stewardship, particularly with PCT, is an important component of the modern approach to VAP treatment duration. It embodies the shift from one-size-fits-all durations to individualized therapy. Along with other diagnostic stewardship measures (rapid pathogen detection, daily clinical evaluations), it helps ensure antibiotics are used no longer than necessary. Next, we address some of those individual patient factors and special pathogen scenarios that may necessitate extending therapy beyond the usual 7 days in selected VAP cases.

## 5. Pathogen-Specific and Patient-Specific Considerations

Although a 7-day course is now standard for VAP in most cases, certain pathogens and clinical scenarios have historically prompted longer treatment [54,55]. Below, we discuss those special considerations, including NF-GNB (e.g., *Pseudomonas* and *Acinetobacter*), carbapenem-resistant organisms, MRSA, and host factors such as immunosuppression or unresolved foci, and what the current evidence suggests about individualizing duration in these contexts.

### 5.1. Non-Fermenting Gram-Negative Bacilli (Pseudomonas *spp.*, Acinetobacter *spp.*, and Stenotrophomonas maltophilia)

VAP caused by NF-GNB, such as *P. aeruginosa* and *Acinetobacter baumannii*, has long been feared to carry a higher risk of relapse or failure with short therapy. Older data indicated a 2-fold higher recurrence of *Pseudomonas* VAP after 8 days versus 15 days of treatment [9]. This led many clinicians to routinely treat *Pseudomonas* for 14 days. However, crucially, mortality did not differ in these early studies. Patients who relapsed after short therapy could be re-treated without worse overall outcomes. The more recent Bouglé 2022 trial also showed a higher recurrence with 8 days of treatment in *Pseudomonas* but, again, no significant mortality or length-of-stay benefit to 15 days of therapy [17]. These findings reinforce a key point, in that extended therapy for *Pseudomonas* may reduce microbiologic relapse, but it does not improve survival, as relapses can typically be managed successfully.

The important question is why *P. aeruginosa* tends to relapse more frequently than other pathogens in VAP. This organism exhibits remarkable adaptive virulence and genomic diversity, allowing for persistence even after initially effective therapy. *P. aeruginosa* can form robust biofilms on endotracheal tubes, tracheostomy hardware, and the respiratory epithelium, creating a protected reservoir that is difficult to eradicate [56,57]. Within these biofilms, bacteria adopt a metabolically quiescent phenotype and are shielded from both antibiotics and host immunity. This facilitates re-seeding of infection once antibiotic pressure is lifted [6,57]. Beyond biofilm formation, *P. aeruginosa* strains differ markedly in serotype- and genotype-specific virulence. Certain serotypes (e.g., O6 and O11) are associated with higher clinical resolution rates, whereas others (O1 and O2) correlate with increased mortality and relapse risk, potentially due to differential expression of type III secretory proteins and other virulence factors [58]. Clonal analyses of ICU isolates further demonstrate that strains responsible for primary colonization (e.g., skin, gut, or upper airway) rarely cause severe pneumonia. In contrast, genetically distinct clones introduced de novo are more often associated with refractory or relapsing VAP [6,59]. After initial antibiotic therapy, residual biofilm-embedded bacteria can re-emerge, manifesting as clinical recurrence despite in vitro susceptibility [60,61]. Distinguishing true relapse from colonization remains challenging but is critical to avoid both overtreatment of benign colonization and undertreatment of persistent infection. *P. aeruginosa* is also notorious for in vivo resistance development, and even brief antibiotic exposure can select less susceptible subpopulations through mutational or adaptive resistance mechanisms [62]. In clinical practice, these biological characteristics translate into longer real-world treatment durations for *Pseudomonas* VAP. Across pivotal RCTs of novel antipseudomonal agents, such as ceftolozane/tazobactam, ceftazidime/avibactam, and cefiderocol, mean treatment durations typically ranged from 7 to 10 days, reflecting persistent clinician concern about relapse risk despite similar short-course outcomes for other pathogens [54]. Many experts, therefore, remain cautious about reducing therapy below 7–8 days for *Pseudomonas*, pending stronger pathogen-specific data. Taken together, the biofilm-forming capacity, clonal heterogeneity, and rapid adaptive potential of *P. aeruginosa* provide a strong biological rationale for its relapse propensity, which should be carefully considered when applying short-course therapy strategies in VAP.

Recent trials and analyses (REGARD-VAP; meta-analyses) suggest that 7–8 days is usually adequate for *Pseudomonas* VAP if the patient improves clinically [10]. Microbiologic relapses were more frequently reported with short courses, but these relapses were typically manageable and not associated with worse patient outcomes [9,10,21]. Both the 2017 European and 2016 US guidelines explicitly state that *Pseudomonas* VAP can be treated for 7 days, conditional on good clinical response [1,2]. They do not recommend automatically extending treatment solely because the infection is caused by *Pseudomonas*. The key is vigilance in a patient with *P. aeruginosa* VAP, and if signs of infection persist or worsen near day 7, one should consider an individualized extension (to 10–14 days) [1,15]. This should go hand in hand with obtaining repeat cultures and imaging to look for uncontrolled infection or complications. Conversely, if a *Pseudomonas* VAP patient defervesces, weans off vasopressors, and improves oxygenation by day 5–7, there is little rationale to continue antibiotics past a week—the evidence indicates it is safe to stop. In summary, short-course therapy is safe for most *Pseudomonas* VAP, but an individualized approach is needed for select cases given the pathogen’s recalcitrance. The clinician should monitor such patients closely following antibiotic discontinuation; if they do relapse, a prompt retreatment will usually still result in a cure.

For *A. baumannii*, data on optimal duration is sparse. Many *Acinetobacter* VAP cases involve MDR or even Carbapenem-resistant *Acinetobacter baumannii* (CRAB). No RCT has specifically focused on short versus long therapy for *Acinetobacter*. Practices vary, with some clinicians defaulting to treatment for ≥14 days, due to *Acinetobacter*’s notorious persistence [63]. However, current guidelines (IDSA and ERS) do not single out *Acinetobacter* as needing longer than 7–8 days if the patient is improving [1,2]. On the other hand, in trials of new drugs for *Acinetobacter*, courses ranged from 7 to 14 days, with many patients treated for >7 days [64,65]. There is a lack of clear evidence that >7 days is beneficial for *Acinetobacter*; thus, most experts will treat a CRAB VAP for ~7 days if the patient shows a clear clinical response. However, they might extend to 10–14 days if the response is slow or if the patient is at high risk, e.g., immunosuppressed [54].

*Stenotrophomonas maltophilia* (*S. maltophilia*) is an intrinsically MDR NF-GNB that classically causes late-onset HAP/VAP, mostly in extensively pretreated ICU patients, including those without overt immunosuppression [66]. Although high-quality randomized duration trials are lacking, contemporary cohort data suggest that outcomes hinge more on timely, active therapy than on extending treatment length by default. In a nationwide ICU study of *S. maltophilia* HAP, predominantly among mechanically ventilated patients, neither antimicrobial modality nor duration was associated with hospital survival. These infections occurred late and in severe, long-stay patients, and empiric regimens were frequently inactive against *S. maltophilia* [67]. In a dedicated VAP cohort, receipt of an active agent, most often trimethoprim–sulfamethoxazole (TMP-SMX), was associated with improved outcomes, whereas longer courses did not confer a mortality advantage [68]. Regarding choice of agent, IDSA’s 2024 AMR Guidance endorses TMP-SMX as first-line therapy for *S. maltophilia*, with levofloxacin or minocycline as reasonable alternatives when TMP-SMX cannot be used. Newer options (e.g., cefiderocol or aztreonam/avibactam) may be considered in resistant or severe disease, often with combination therapy per susceptibility and clinical status [69]. Observational comparative-effectiveness studies indicate similar mortality with levofloxacin versus TMP-SMX in bloodstream and lower-respiratory infections caused by *S. maltophilia*, supporting these alternatives when appropriate [70]. Importantly, a recent propensity-matched analysis focused on *S. maltophilia* pneumonia found that timely initiation (≤48 h) of an active agent significantly improved survival and clinical outcomes versus delayed therapy. This highlights that prompt activity outweighs the impact of prolonged duration [71]. In the absence of randomized duration trials for *S. maltophilia* VAP, authoritative references and synthesis of cohort data support a ~7-day total course in immunocompetent patients who demonstrate clear clinical improvement by day 5–7. This aligns with the broader VAP paradigm in this review. Extension to 10–14 days is reasonable for slow clinical response, profound immunosuppression, bacteremia, or complicated pulmonary foci or when effective therapy was delayed (i.e., time to first active agent) [68]. In practice, we suggest adopting the same “extend with purpose” rules used for other pathogens in this manuscript: discontinue therapy at approximately 1 week when infection control and improvement are evident, and justify and reassess any extension at 48 h intervals, based on objective signs of ongoing infection and source control considerations. If the patient is clinically improving on an active regimen, a ~7-day course is usually sufficient; prolong treatment only with explicit clinical justification [68].

### 5.2. Carbapenem-Resistant and Difficult-to-Treat Organisms

VAP caused by highly resistant Gram-negative pathogens, such as carbapenem-resistant *Enterobacterales* (CRE), CRAB, or the so-called “difficult-to-treat resistance” (DTR) *P. aeruginosa,* poses a major clinical challenge [72]. These infections require newer antibiotic agents with limited data or salvage antibiotics (e.g., tetracycline derivatives, polymyxins, aminoglycosides, and fosfomycin) [69,73,74]. One might intuitively believe that such tough pathogens require prolonged treatment for eradication. However, no randomized controlled trial (RCT) has demonstrated that extending therapy beyond a week improves outcomes in these cases. This could be due to the difficulties in studying resistant pathogens, especially in the context of high patient variability and treatment response. The REGARD-VAP study, which included patients with highly resistant infections (about one-third with carbapenem-resistant bacteria), found that a 7-day treatment course was non-inferior to longer therapy, even for this subgroup. While this does not prove that every case of CRE/CRAB/DTR VAP is cured within 7 days of treatment, it suggests that many can be successfully treated within this timeframe [10]. Granted, these were patients who met criteria for clinical improvement, so it does not prove that every CRE/CRAB/DTR VAP is cured by 7 days, but it does indicate that many can be, and treating longer did not yield better results for those who improved by day 7. The current approach is generally to aim for 7 days in CRE/CRAB/DTR VAP if the patient is clearly improving but to have a lower threshold to extend if there are any complicating factors [1,64]. “Difficult cases” that might justify 10–14 days include persistent fever/inflammation at the end of week 1, inadequate source control (e.g., unresolved abscess or empyema), concerns about drug penetration (i.e., the selected agent achieves suboptimal lung concentrations), or if therapy was delayed initially. Both Chinese and German guidelines mention that MDR/DTR pathogens may necessitate extended therapy on a case-by-case basis, essentially leaving it to clinician judgment [14,15]. It is notable that in trials of novel antibiotics for CRE/CRAB/CRPA (e.g., cefiderocol, ceftazidime-avibactam), protocols often allowed for up to 10–14 days, and many patients did receive >7 days [75,76,77]. This reflects the reality that practitioners were not uniformly comfortable stopping at 1 week in those studies, likely due to severity and lack of precedent. Until better evidence emerges, an individualized duration is prudent for these organisms: start with the intention of a short course, but be prepared to extend if required. Conversely, if a patient with CRE/CRAB/DTR VAP improves in 5–7 days, the shorter antibiotic treatment duration of 7 days will likely suffice. The key is continuous evaluation of the patient’s trajectory and thereby individualizing these decisions for each patient.

### 5.3. Methicillin-Resistant Staphylococcus aureus VAP

For methicillin-resistant *Staphylococcus aureus* (MRSA) pneumonia, one might assume longer therapy is needed given its virulence. Interestingly, there is little evidence that MRSA VAP inherently requires more than 7 days. Historically, MRSA pneumonia (HAP and VAP) has been treated for 7–10 days except when complicated by bacteremia [78,79]. The IDSA guidelines do not recommend extending VAP treatment solely due to MRSA; they apply the same 7-day rule as for other pathogens, provided the patient improves [1]. The unique issue with *S. aureus* is the propensity for bacteremia and metastatic infection. About 10–20% of VAP cases can have concurrent bacteremia, and MRSA is one of the more common causes of VAP-associated bloodstream infection [80]. If MRSA VAP is accompanied by MRSA bacteremia, then the patient effectively has MRSA sepsis, and the standard of care for MRSA bacteremia is at least 14 days of IV therapy (from the first negative culture) [79]. In such cases, therapy must be extended for 2 weeks or more because of the risk of endovascular infection and metastatic seeding, as the pneumonia is only one part of a systemic infection. Thus, any VAP with *S. aureus* bacteremia is a special case, and the MRSA bacteremia guidelines (usually 14 days minimum) should be followed [1,2,81]. On the other hand, if MRSA VAP is confined to the lungs (no bacteremia; no empyema), data indicates that 7–8 days of linezolid or vancomycin are sufficient. One retrospective study found that the average duration of treatment for MRSA VAP in practice was ~10.5 days. This suggests clinicians often err on the longer side, yet outcomes did not differ between those treated for >8 days vs. ≤8 days [82]. Another analysis noted that patients with bacteremia VAP had worse mortality outcomes than non-bacteremia, but the duration of therapy used was similar in both. This implies that many doctors were not extending courses even in bacteremia cases at that time, or if they did, it did not significantly change outcomes [83]. The take-home point is as follows: treat MRSA VAP for 7 days if the patient improves and there is no concurrent invasive infection. If MRSA bacteremia is present, however, longer treatment (≥14 days) is required along with a thorough search for endocarditis or other metastatic foci. The same principle applies to methicillin-susceptible *Staphylococcus aureus* (MSSA), where bacteremia cases need prolonged therapy; non-bacteremia pneumonia alone can be treated with a 7-day course. Always tailor to the patient: for example, if MRSA VAP has caused lung cavitations or abscesses, it might warrant a longer course due to the complexity of those lesions (similar to how abscesses are generally managed). But routine extension “just because it is MRSA” is not supported by evidence or guidelines.

### 5.4. Immunocompromised Patients

A population that consistently falls outside trial data is severely immunosuppressed patients (neutropenic, bone marrow transplant, on high-dose steroids, advanced HIV, etc.). Virtually all RCTs on VAP (Chastre, REGARD, etc.) excluded immunocompromised patients [9,10]. As a result, there is no direct evidence supporting short antibiotic courses in this group. Many experts adopt a more cautious approach in immunosuppressed patients, reasoning that impaired host defenses might require a longer antibiotic course to fully clear infection. For instance, in a neutropenic patient with VAP, clinicians might opt for 10–14 days by extrapolating from how febrile neutropenia infections are treated, even though evidence is widely lacking. Guidelines generally acknowledge the lack of data and suggest individualizing treatment. The ERS 2017 guideline, for example, noted that immunocompromised hosts could be an exception where a longer course of therapy might be considered [2]. Until further research addresses this question, clinicians often “err on the side of caution” and opt for longer treatment in profoundly immunocompromised VAP patients. However, unnecessary prolongation should still be avoided. A reasonable compromise is to start with a 7–8-day course, and if the patient is not clearly improving, consider extending therapy for a few more days while continuing to monitor the patient’s progress. In summary, a short course should be used where possible, but a lower threshold for extending therapy is appropriate for patients with major immune deficits, given the uncertainty surrounding this patient population.

While a 7-day antibiotic course is sufficient for most patients with ventilator-associated pneumonia (VAP), the clinical scenarios discussed above may justify extending therapy. These decisions should always be guided by the overall clinical trajectory, microbiological data, and source control. Table 2 summarizes common situations in which extended treatment seems appropriate, along with the underlying rationale and typical duration. Whenever possible, the need for prolonged therapy should be explicitly justified and re-evaluated daily in the context of the patient’s clinical improvement and infection control.

## 6. When to Consider Extending Therapy Beyond 7 Days

Despite the overall push toward shorter regimens, certain clinical scenarios do warrant extended antibiotic treatment for VAP. Both guidelines and expert opinion recognize that a subset of patients may benefit from more than 7 days. The guiding principle is “do not extend by default; extend with purpose”, thus requiring a clear reason to continue antibiotics past 1 week. Below are situations where therapy beyond 8 days may be justified:

- Lack of clinical improvement by day 5–7: If a patient is not responding to therapy as the 1-week mark approaches (e.g., if they remain febrile, are still on high ventilator support, or are showing no improvement in oxygenation), continuation of the antibiotics with reassessment of the regimen is prudent [84]. Failure to improve raises concerns about inadequate source control, inappropriate antibiotic choice (e.g., due to a resistant pathogen or inadequate coverage), or complications. In such cases, stopping treatment at 7 days would be risky since the infection may not be cleared. Thus, persistent clinical signs of infection at the end of a short course warrant extension until improvement is seen and there is a rigorous evaluation of why the response is slow [84].
- Persistently high PCT and/or inflammatory markers: If biomarkers like PCT remain elevated or show minimal decline by day ~7, it may indicate ongoing infection [85]. Many PCT-guided protocols suggest not stopping antibiotics if PCT is still >0.5 ng/mL or does not decline by ~80% of its peak value [26,30,86]. For instance, a PCT that is minimally reduced by day 7 (e.g., from 5 to 4) may indicate insufficient infection control, suggesting the need for continued therapy. While PCT levels should only be interpreted in a clinical context (e.g., renal failure can keep PCT elevated), a stagnant or rising PCT by day 5–7 could justify prolonged therapy or at least prompt further investigation before stopping. Conversely, a low PCT despite clinical deterioration may indicate a nonbacterial cause (e.g., an organizing pneumonia). Thus, trends in PCT or other inflammatory markers can support decisions regarding treatment extension. Persistently elevated inflammatory markers in the absence of clinical improvement suggest ongoing infection and support the continuation of antibiotic therapy with reassessment.
- MDR/XDR pathogen with slow response: In cases where the causative pathogen is a difficult MDR (like CRAB, CRE, or DTR *Pseudomonas*) and the patient’s clinical improvement is slow, a longer course of treatment (e.g., 10–14-day course) may be warranted [17,21,69]. For example, suppose a patient with carbapenem-resistant *Acinetobacter* VAP is on appropriate therapy but by day 7 remains febrile and has purulent secretions—many would continue treatment given the pathogen’s recalcitrance. In REGARD-VAP, patients with MDR organisms did well with short courses if they improved. However, in practice, if improvement is lacking, clinicians extend therapy for these pathogens while monitoring closely [10]. Essentially, MDR VAP accompanied by a slow clinical resolution will likely require extension beyond 7 days in a case-by-case decision.
- Unresolved or complicated infection foci: Certain pulmonary complications inherently require longer treatment. Examples include lung abscesses, necrotizing pneumonia, empyema, or cavitating lesions [15,87]. These conditions often take more time to sterilize and sometimes necessitate adjunct procedures (drainage; surgery). Guidelines recommend extended therapy if an empyema cannot be fully drained, and the German guidelines for 2024 specifically advise prolonging therapy in such cases [15]. Similarly, necrotizing pneumonia or extensive lung destruction might clear more slowly. If imaging reveals a cavity or abscess, treatment is often extended beyond 7 days, with some cases requiring 3–4 weeks for abscesses, although data is scant in VAP-related abscesses [88]. In summary, unresolved foci mean an unresolved infection, necessitating continued therapy as well as attempts at source control.
- Bacteremia or extrapulmonary spread: Any VAP episode accompanied by bacteremia, especially with organisms like *S. aureus*, generally warrants extending treatment [79]. In cases of bloodstream infection, antibiotics are typically continued for 10–14 days, depending on the organism, to ensure complete clearance from the blood and prevent metastatic seeding [79,83]. For instance, VAP with MRSA bacteremia necessitates at least 14 days per MRSA guidelines [79]. However, this dogma is also under debate, as a recent RCT elucidates that even in an uncomplicated Gram-negative bloodstream infection, 7 days of antibiotic treatment seems sufficient [80]. Likewise, if the pneumonia has seeded to other sites (septic emboli to other organs, septic arthritis, etc.), the total duration must cover treatment of those secondary sites. Essentially, once infection is systemic or outside the lungs, it is no longer just “VAP”, but rather, it is a disseminated infection requiring a longer course. Always follow up blood cultures and consult relevant guidelines for bacteremic pneumonias.
- Immunosuppression: Although evidence is lacking, as noted, many clinicians err on longer treatment in immunocompromised patients [89]. For example, in a neutropenic leukemia patient with VAP, one might treat for 10–14 days since their neutrophils (key for bacterial clearance) are low [90]. The rationale is that in an immune-weakened host, the clearance of bacteria may be slower even if on appropriate antibiotics, so additional days of therapy might help prevent relapse once antibiotics are stopped. This is more of an expert-opinion stance rather than evidence-based, but it is commonly practiced. Importantly, one should also accelerate supportive measures to restore immune function (e.g., granulocyte colony-stimulating factor if neutropenic; reducing immunosuppressants if feasible) because antibiotics alone might not suffice if the immune system is dampened.

Whenever an extension of therapy is considered, it is critical to re-evaluate the entire treatment strategy rather than simply adding more days. Continuing the same antibiotic regimen in a patient who is not improving without reassessing or modifying management is often futile and may be harmful. Key questions to ask at day 5–7 if considering an extension: Is my antibiotic coverage appropriate? Do culture results suggest we missed an organism? Should we broaden or adjust drugs? Is there adequate source control? Does the patient have an undrained abscess, infected line, or other focus? Could there be a complication or alternate diagnosis?

It may be indicated to obtain repeat cultures, repeat imaging (e.g., a chest CT), or even perform a new bronchoscopy to identify why the patient is not improving. Simply adding more days of the same antibiotics without answering these questions could lead to worse outcomes. For example, if the real issue is a ventilator-associated tracheobronchitis or an atelectasis rather than active pneumonia, continuing antibiotics is of limited benefit. If an abscess is present, drainage may be indicated in addition to prolonged antibiotic therapy. Thus, any extension beyond the standard course should trigger a thorough work-up of “why has this patient not gotten better?” The mantra is “treat until the patient is better and infection is controlled.” For most patients, this equates to about 1 week; for others who have not improved by this time point, therapy should be continued while the underlying reason is investigated. Every day beyond day 7 should be justified by a specific clinical need, as outlined above. This patient-tailored approach maximizes the benefits of antibiotics while minimizing potential harms.

## 7. The Evolving Stewardship Paradigm and Future Directions

The trend toward shorter VAP treatment reflects a broader shift in critical care infectious disease management, moving from “maximally aggressive” antibiotic use to “maximally effective but parsimonious” use [54]. We now recognize that less can be more: in many cases, shorter antibiotic courses achieve the same outcomes while reducing toxicity and resistance pressures. However, implementing this paradigm requires careful balancing and cultural change in the ICU.

A central concern is a classic fear: will shorter therapy lead to more relapses? Opponents of short courses worry about undertreatment and recurrence of pneumonia once antibiotics are stopped. Indeed, as reviewed, certain pathogens (i.e., *Pseudomonas*) do have somewhat higher relapse rates with abbreviated therapy [9,17,18,21]. But the counterargument, supported by trials and meta-analyses, is that such relapses are usually treatable and do not worsen long-term outcomes [9,17,18,21]. Meanwhile, the harms of prolonged therapy are guaranteed for every patient: each extra day of antibiotics brings risk of drug toxicity (renal failure, liver injury, etc.) and *C. difficile* infection and promotes antimicrobial resistance in the patient and the ICU flora [1,91]. From a risk–benefit perspective, short courses clearly have an advantage for the majority. We must acknowledge, however, that ICU patients with VAP represent a heterogeneous cohort. There is likely a subset, perhaps those with very high bacterial burden, extensive lung damage, or severely impaired host defenses, who might genuinely benefit from a longer course of therapy to achieve complete infection eradication [1,2,10]. This is where individualized clinical judgment remains crucial: not every VAP should automatically stop at day 7 if the patient’s course dictates otherwise. Factors like persistent fever, ongoing high ventilatory requirements, or new complications signal caution and possibly a need for extension. The goal is to catch those who need longer therapy without overtreating the rest.

Another key aspect is the role of diagnostic stewardship and follow-up in enabling shorter durations. If we are going to treat for only 5–7 days, we must be confident in our diagnosis and vigilant for recurrence. VAP diagnosis is notoriously tricky, and overdiagnosis is common, as many patients are treated for VAP based on nonspecific signs when they might not truly have bacterial pneumonia [92,93]. Thus, one strategy to shorten therapy is to ensure that the patient truly has VAP in the first place. If a patient is started on antibiotics for suspected VAP, and by day 3, cultures are negative and clinical suspicion wanes, stopping antibiotics early (3–5 days) should be considered [1,93]. This prevents treating a non-infectious cause for a full week or two; this achieves an aspect of diagnostic stewardship. Conversely, if a patient truly has VAP and we stop at 7 days, we need a plan to monitor for relapse. This usually means close clinical observation with frequent respiratory assessments, perhaps follow-up chest X-ray or ultrasound in high-risk cases. Antibiotic “time-outs” at 48–72 h are a great practice: formally review culture results and patient status at day 3 to ensure appropriate narrowing or discontinuation (Figure 1). Studies have shown that ICUs implementing such reassessment protocols can reduce antibiotic days of therapy without worsening clinical outcomes [94].

The emergence of standardized definitions for VAP recurrence (such as the proposed RECUVAP criteria) will also help, both in research and practice [24]. If we can clearly define what constitutes a relapse versus a new infection versus colonization, clinicians can feel more secure in stopping therapy and monitoring for specific signs of recurrence. It will also help future studies identify who truly failed short therapy and why.

The prerequisite for shorter therapy is optimal initial management [95,96]. Obtaining the antibiotic selection and dose right from day 1 and achieving source control if needed are critical “front-loaded” steps. If this is performed correctly, the infection is knocked down rapidly, and short therapy succeeds. Inadequate initial empiric therapy, on the other hand, is known to worsen outcomes. Guidelines note that if the empiric regimen was inappropriate, one might consider a longer duration once effective therapy is started [1,2]. This is because there was a period of uncontrolled infection at the beginning that could have allowed a larger bacterial burden or complications. In essence, a short-course strategy cannot compensate for undertreatment early on. We must both start right and stop early. “Start right” means empiric therapy should be broad enough to cover likely MDR in high-risk patients (and then de-escalate once cultures return). Ensuring adequate drug levels, through optimized dosing, prolonged infusions for beta-lactams, and therapeutic drug monitoring if available, can also ensure we enhance antimicrobial efficacy. Achieving the right dose at the infection site is as critical as choosing the right agent. Inadequate early drug exposure, especially in critically ill, hyperdynamic, or edematous patients, can lead to subtherapeutic concentrations in the epithelial lining fluid. This can result in early treatment failure or relapse despite adequate nominal dosing [97,98]. This pharmacokinetic challenge is particularly relevant for hydrophilic agents such as β-lactams, aminoglycosides, and glycopeptides, whose distribution volumes and clearance are profoundly altered in sepsis and during mechanical ventilation [97,99,100]. Consequently, modern stewardship increasingly emphasizes “hit hard, hit fast”: administer an appropriate empirical regimen at optimized doses and administration modes (e.g., loading doses, extended or continuous β-lactam infusions, and therapeutic drug monitoring where feasible) to achieve rapid and sustained bactericidal exposure at the infection focus [101,102,103,104]. When drug penetration and early target attainment are ensured, clinicians can safely shorten treatment without increasing recurrence risk. This is a concept supported by pharmacodynamic modeling and clinical outcome data linking early adequate exposure to improved survival in VAP and sepsis [104,105,106,107]. In parallel, the risk of initial inappropriate therapy remains a practical reality in MDR-endemic ICUs. Early use of rapid syndromic molecular panels and locally adapted diagnostic algorithms can mitigate this risk by accelerating identification of pathogens and resistance determinants, enabling earlier de-escalation or switch to an active agent [43,108,109]. However, such tools must be interpreted in light of local flora and resistance mechanisms: for example, *P. aeruginosa* often exhibits non-enzymatic resistance (e.g., efflux or porin loss) not detected by standard panels, underscoring the need for clinical judgment and site-specific validation [110,111]. Integrating rapid diagnostics with pharmacokinetic/pharmacodynamic (PK/PD)-optimized dosing thus represents the “precision front-end” of the short-course paradigm: start right, hit hard, and then stop early. With that solid initial management, we increase the success of a short course and reduce the chance that a hidden reservoir lingers.

Finally, there is the challenge of changing ICU culture and clinician habits. For decades, more antibiotics (longer and broader) were equated with better care in severe infections. Breaking this mindset requires education and system-level support. Notably, the recent large, multinational (72 countries) D-PRISM survey that aimed to assess practices in diagnosis and management of pneumonia in the ICU found that, overall, 47% of ICU clinicians still favor >7-day antibiotic courses for VAP [16]. Higher national income status was associated with shorter VAP treatment duration: 39.1%, 54.9%, and 57.2% of clinicians indicated a >7-day intended duration in higher-, upper-/middle-, and low-/low–middle-income countries, respectively [16]. Other factors that encouraged shorter course adoption included formal ICU training, having antibiotic stewardship programs, and routine ‘time-outs’. These results imply that institutional protocols and nudges can influence practice. Many hospitals that have successfully implemented short-course antibiotic treatment have introduced automatic stop orders or require documented justification to continue antibiotics beyond a predefined duration. Such measures enhance critical, deeper thinking and support a more thorough assessment of the patient’s condition, ensuring decisions to continue antibiotics for VAP are based on objective findings and evidence rather than driven by risk aversion.

## 8. Conclusions

VAP remains a serious ICU infection, but our approach to its antibiotic management has evolved significantly. Evidence now shows that short-course therapy (~7 days) is sufficient for most VAP patients [10,11,17,18,21,54]. Shorter treatment reduces patient harm (toxicity, resistance, and costs) without compromising effectiveness [9,20]. Consequently, current guidelines advocate 7- to 8-day regimens for uncomplicated VAP, reserving longer courses only for defined scenarios such as poor clinical response or uncontrolled foci [1,2,15]. The modern paradigm emphasizes an individualized, response-driven duration rather than a one-duration-fits-all policy [54]. Clinicians are encouraged to reassess VAP therapy daily, especially at 48–72 h and around day 7, to decide if antibiotics can be stopped (Figure 1). Tools like PCT trending, clinical assessment tools, and routine stewardship “time-outs” support these decisions, providing objective reassurance when it is time to discontinue therapy. Importantly, stopping antibiotics should be considered once the patient’s infection is adequately controlled. This is often reflected by defervescence, improved respiratory status, and downward trends in inflammatory markers. For special cases such as VAP with bacteremia, abscess, or immunosuppression, clinicians may need to extend treatment judiciously while addressing underlying issues (e.g., source control, immune support). Even then, the extension should not be indefinite: it should be for a defined purpose and duration, with frequent re-evaluation. In all cases, a conscious balance must be struck between the slight risk of recurrence if stopping too early and the definite risks of continuing antibiotics too long.

Future antibiotic strategies for VAP should continue to shift toward response-adapted rather than pathogen-driven duration, particularly for infections caused by NF-GNB or resistant organisms. In these settings, treatment extension should be explicitly justified by ongoing clinical instability or objective evidence of uncontrolled infection, not by pathogen identity alone. At the same time, emerging tools such as rapid diagnostics, molecular resistance profiling, and electronic decision-support systems may further refine antibiotic selection, enable earlier de-escalation, and support safe discontinuation. Finally, sustained reductions in VAP-related morbidity and antimicrobial overuse will require alignment between bedside clinicians, stewardship teams, and institutional leadership. Ongoing education, audit-and-feedback mechanisms, and integration of evidence-based duration targets into ICU protocols are essential to closing the gap between evidence and practice.

Looking forward, effective control of VAP at the hospital level will increasingly depend on structured, system-wide implementation of antimicrobial stewardship principles rather than on individual prescribing decisions alone. Hospitals should aim to embed standardized reassessment strategies, such as mandatory antibiotic “time-outs” at 48–72 h and near day 7, into routine ICU practice, coupled with clear accountability for stop-or-extend decisions. Diagnostic stewardship, including timely respiratory sampling, judicious use of biomarkers, and avoidance of unnecessary empiric prolongation in culture-negative cases, will be central to preventing overtreatment.

Ultimately, the management of VAP duration is transitioning from an era of “more is better” to an era of “optimal is better”, delivering enough antibiotics to cure pneumonia but not so much that this creates further complications. The evidence reviewed in this manuscript underlines that, for most patients, “enough” is about 1 week. By adhering to the latest evidence and recommendations, intensivists can confidently treat VAP with shorter courses in appropriate patients, using clinical and diagnostic insights to guide therapy duration. This approach will maximize patient benefit by curing infection while minimizing the collateral damage of antibiotics. Ongoing vigilance, however, remains necessary both at the bedside (to identify patients who require longer therapy) and at the stewardship level (to ensure the consistent application of these practices). With thoughtful implementation, the evolving paradigm of shorter-duration treatment in VAP stands to improve patient outcomes and antimicrobial use in ICUs moving forward.

## Figures and Tables

**Table 1:** Guideline recommendations on antibiotic duration for VAP.

Guideline (Year)	Recommended Duration	Notable Caveats/Comments
IDSA/ATS (USA, 2016); [1]	7 days (preferred)	Strong recommendation. Longer therapy only if evidence of ongoing infection despite 7 days. Emphasizes reducing antibiotic exposure.
ERS/ESICM/ESCMID (Europe, 2017); [2]	7–8 days (conditional recommendation)	Include difficult pathogens in 7–8d course if clinically improving. Extend duration if poor clinical response, immunocompromised host, or complications (e.g., empyema).
French HAP Guideline—SFAR (2018); [13]	7 days (strong recommendation)	Similar to IDSA; based on evidence that 7d is generally sufficient. Acknowledges *Pseudomonas* may relapse more often with short course, but no survival difference.
China Thoracic Society (2018); [14]	7–10 days (typical range)	Usually, 7d adequate; up to 10d often used in practice. Extend past 10d if slow improvement, MDR pathogen, or immune deficit. Recommends adjunct PCT monitoring to inform duration.
German National Guideline—DZIF/DGIIN (2024); [15]	Approx. 7 days (standard for VAP)	Reiterate 7d if clinical response. For unresolved foci (e.g., undrained pleural infection) or ongoing signs of infection at day 7, consider longer (10–14d). Suggests biomarkers (PCT) can aid decisions in equivocal cases.

IDSA—Infectious Diseases Society of America; ATS—American Thoracic Society; ERS—European Respiratory Society; ESICM—European Society of Intensive Care Medicine; ESCMID—European Society of Clinical Microbiology and Infectious Diseases; SFAR—French Society of Anesthesia & Resuscitation; DZIF—German Center for Infection Research; DGIIN—German Society of Internal Intensive Care.

**Table 2:** Clinical scenarios where extended antibiotic treatment may be justified in VAP.

Clinical Scenario	Rationale for Extended Therapy	Typical Duration	Level of Evidence
Slow or incomplete clinical response (persistent fever, elevated inflammatory markers)	Indicates delayed infection control or alternative foci	10–14 days, reassess every 48 h	Expert consensus
Bacteremia or endocarditis due to the VAP pathogen	Requires systemic source control and ensures adequate sterilization	10–14 days, depending on source	Evidence-based
Empyema, abscess, or necrotizing pneumonia	Reduced antibiotic penetration and higher bacterial load	≥14 days, individualized	Evidence-based
Inappropriate initial empiric therapy	Delayed pathogen control increases recurrence risk	Count duration from first active therapy	Expert consensus
Infections with difficult-to-treat or high-risk pathogens (e.g., *Pseudomonas aeruginosa*, *Acinetobacter baumannii*, MRSA, NF-GNB)	Potential for persistence and biofilm formation	10–14 days depending on course	Evidence-based
Severe immunosuppression (neutropenia, transplantation, high-dose steroids, advanced malignancy)	Impaired host defence; slower bacterial clearance	≥10 days or until immune recovery	Expert consensus
Uncontrolled or ongoing infectious focus (e.g., unremoved device, open chest, uncontrolled drainage)	Ongoing inoculum prevents eradication	Continue until source control achieved	Expert consensus

Selected clinical constellations in which antibiotic treatment beyond the standard 7-day course may be appropriate. Duration should always be individualized according to clinical response, source control, and host factors. Prolongation is justified only when infection control is uncertain or delayed; reassessment is recommended at least every 48 h.

## Data Availability

No new data were created or analyzed in this study. Data sharing is not applicable to this article.

## Abbreviations

The following abbreviations are used in this manuscript:

*A. baumannii*	*Acinetobacter baumannii*
AMR	Antimicrobial resistance
ATS	American Thoracic Society
BAL	Bronchoalveolar lavage
CPIS	Clinical Pulmonary Infection Score
CPIS-PLUS	Clinical Pulmonary Infection Score plus procalcitonin and lung ultrasound
CRAB	Carbapenem-resistant *Acinetobacter baumannii*
CRE	Carbapenem-resistant *Enterobacterales*
CRP	C-reactive protein
CRPA	Carbapenem-resistant *Pseudomonas aeruginosa*
CT	Computed tomography
DGIIN	German Society of Internal Intensive Care Medicine
DTR	Difficult-to-treat resistance
DZIF	German Center for Infection Research
ERS	European Respiratory Society
ESCMID	European Society of Clinical Microbiology and Infectious Diseases
ESICM	European Society of Intensive Care Medicine
HAP	Hospital-acquired pneumonia
HIV	Human immunodeficiency virus
ICU	Intensive care unit
IDSA	Infectious Diseases Society of America
IL-6	Interleukin-6
LUS	Lung ultrasound
MDR	Multidrug-resistant
MRSA	Methicillin-resistant *Staphylococcus aureus*
NF-GNB	Non-fermenting Gram-negative bacilli
*P. aeruginosa*	*Pseudomonas aeruginosa*
PCT	Procalcitonin
PK/PD	Pharmacokinetics/pharmacodynamics
RCT	Randomized controlled trial
*S. aureus*	*Staphylococcus aureus*
*S. maltophilia*	*Stenotrophomonas maltophilia*
SFAR	French Society of Anesthesia and Resuscitation
TMP-SMX	Trimethoprim–sulfamethoxazole
VAP	Ventilator-associated pneumonia
XDR	Extensively drug-resistant
